# Pravastatin reduces plasma levels of extracellular vesicles in pregnancies at high risk of term preeclampsia

**DOI:** 10.3389/fphar.2023.1166123

**Published:** 2023-06-22

**Authors:** Jean Michell Santoyo, José Antonio Noguera, Francisco Avilés, Trinidad Hernández-Caselles, Catalina de Paco-Matallana, Juan Luis Delgado, Santiago Cuevas, M. Teresa Llinás, Isabel Hernández

**Affiliations:** ^1^ Department of Physiology, Institute of Biomedical Research (IMIB-Pascual Parrilla), University of Murcia, Murcia, Spain; ^2^ Institute of Biomedical Research (IMIB-Pascual Parrilla), University Clinical Hospital Virgen de la Arrixaca, Murcia, Spain; ^3^ Department of Biochemistry and Molecular Biology “B” and Immunology, Institute of Biomedical Research (IMIB-Pascual Parrilla), University of Murcia, Murcia, Spain; ^4^ Molecular Inflammation Group, Institute of Biomedical Research (IMIB-Pascual Parrilla), University Clinical Hospital Virgen de la Arrixaca, Murcia, Spain

**Keywords:** preeclampsia, endothelial dysfunction, statins, pravastatin, cardiovascular disease, extracellular vesicles

## Abstract

**Introduction:** Elevated plasma levels of extracellular vesicles have been associated with impaired placentation, angiogenesis imbalance, intravascular inflammation, and endothelial dysfunction in women with preeclampsia, thus suggesting that circulating vesicles may be a good therapeutic target for the treatment of the disease. Recently, statins have been considered a potential treatment for the prevention of preeclampsia because of their pleiotropic effects, including the improvement of endothelial dysfunction and inhibition of inflammatory responses. However, the effects of these drugs on circulating vesicles concentration in women at risk of preeclampsia have not been established. Herein, we aimed to assess the effects of pravastatin on circulating extracellular vesicle generation in women at high risk of term preeclampsia.

**Methods:** In a sample of 68 singleton pregnant women participating in the multicenter, double-blind, placebo-controlled STATIN trial (Nº EducraCT 2016-005206-19 ISRCTN), 35 women received a placebo and 33 women received a 20 mg/day dose of pravastatin for approximately 3 weeks (from 35 to 37 weeks of gestation until delivery). Large extracellular vesicles were characterized and quantified by flow cytometry using annexin V and cell-specific antibodies directed against platelet, endothelial, leukocyte, and syncytiotrophoblast cell surface markers.

**Results:** In women who received the placebo, a significant increase in the plasma levels of large extracellular vesicles from platelets (34%, *p* < 0.01), leukocytes (33%, *p* < 0.01), monocytes (60%, *p* < 0.01), endothelial cells (40%, *p* < 0.05), and syncytiotrophoblast cells (22%, *p* < 0.05) were observed. However, treatment with pravastatin significantly reduced the plasma levels of large extracellular vesicles from platelets (42%, *p* < 0.001), leukocytes (25%, *p* < 0.001), monocytes (61%, *p* < 0.001), endothelial cells (69%, *p* < 0.001), activated endothelial cells (55%, *p* < 0.001), and syncytiotrophoblast cells (44%, *p* < 0.001).

**Discussion:** These results indicate that pravastatin reduces the levels of activated cell-derived membrane vesicles from the maternal vasculature, blood, and placental syncytiotrophoblast of women at high risk of term preeclampsia, suggesting that this statin may be beneficial in reducing endothelial dysfunction and pro-inflammatory and pro-coagulatory state characteristics of the disease.

## Introduction

Preeclampsia (PE) is one of the most common pregnancy complications, affecting 3%–8% of pregnant women worldwide, and is a leading cause of fetal and maternal morbidity and mortality (Khan et al., 2006; Ghulmiyyah and Sibai, 2012; ACOG Practice Bulletin, 2020). Although the etiology of PE is still widely debated, it is now generally accepted that the disease results from placental dysfunction, mainly associated with defective placentation in the case of early PE (before 34 weeks of gestation) or with poor uteroplacental perfusion, related to previous maternal cardiovascular and metabolic alterations, in late PE (≥34 weeks of gestation) (Poon et al., 2019). PE is characterized by an imbalance of circulating angiogenic factors, excessive inflammation, and endothelial dysfunction, ultimately leading to clinical manifestations such as hypertension, proteinuria, and end-organ damage (Rana et al., 2019). These changes can persist after pregnancy and manifest as cardiovascular disorders later in life. In this regard, it has been suggested that endothelial damage caused by PE persists into postnatal life and increases the risk of future cardiovascular diseases (CVDs) (Newstead et al., 2007).

Several research groups have reported high levels of circulating microvesicles (MVs) in the plasma of women with PE. Increased concentrations of MVs derived from platelets, syncytiotrophoblasts, leukocytes, monocytes, and endothelial cells have been found in women with PE compared with normal pregnant women (Meziani et al., 2006; Lok et al., 2008a; Alijotas-Reig et al., 2012; Chen et al., 2012; Marques et al., 2013). Extracellular vesicles (EVs) play a dynamic role in the communication between the placenta and maternal vascular cells (vascular endothelium, leukocytes, and platelets) that under normal conditions contribute to the normal evolution of gestation by adapting the maternal vasculature. Depending on maternal predisposing factors or placental abnormalities, some of these vascular EVs may be capable of initiating a cascade of events, leading to the development of PE (Alijotas-Reig et al., 2012; Mikhailova et al., 2014). Maternal conditions associated with endothelial cell activation and immune system modulation may trigger the release of EVs that increase the processes of inflammation, coagulation, and endothelial dysfunction (Alma et al., 2018). It has been reported that the concentration of syncytiotrophoblast-derived EVs in the maternal circulation directly reflects the state of placental injury (Reddy et al., 2008; Chen et al., 2012) and is associated with the severity of hypertension in preeclamptic women (Lok et al., 2008a; Chen et al., 2012). Furthermore, EVs isolated from preeclamptic women have been implicated in trophoblast dysfunction, placentation abnormalities, imbalanced angiogenesis, and intravascular inflammation and have been reported to activate endothelium, monocytes, and platelets (Wang et al., 2021), thus suggesting that they may be a good therapeutic target in PE. In this sense, several studies have shown that statins decrease the elevated levels of circulating MVs of different cellular origins in individuals with hyperlipidemia (Suades et al., 2013), ischemic cardiomyopathy (Huang et al., 2012), and diabetic patients with chronic kidney disease (Inglis et al., 2015), pathologies that share mechanisms of endothelial dysfunction with PE.

Over the past decade, data on the safety of statins in pregnancy (Costantine et al., 2016), and the demonstrated effects of these drugs on reducing endothelial dysfunction, inflammation, and immunomodulation (Rao et al., 2022), have led to an increase in clinical trials using them as preventive therapy to treat term PE. However, the effectiveness of these drugs in preventing the disease remains controversial. Understanding the effect of statins on EV levels in women at high risk of term PE may be relevant to evaluating the efficacy of these drugs for the prevention of the disease. Therefore, the goal of the present study is to assess the effects of pravastatin treatment on the levels of circulating EVs and their cellular origin in women at high risk of term PE.

## Methods

This is a prospective, descriptive, and analytical study of the effect of treatment with pravastatin on the levels of circulating large extracellular vesicles (LEVs) in pregnant women at risk of term PE, which is developed in the framework of a randomized, double-blind, placebo-controlled, and multicenter clinical trial (STATIN, EC Nº EducraCT 2016-005206-19, ISRCTN16123934, https://doi.org/10.1186/ISRCTN16123934) and conducted to examine the prophylactic use of this drug, starting at 35–37 weeks of gestation, in reducing the incidence and severity of PE at term. Eligible participants were randomly selected through a term PE screening study conducted among women attending for their routine hospital visit at Virgen de la Arrixaca University Clinical Hospital (HCUVA) with 35^+0^–36^+6^ weeks of gestation from December 2018 to November 2019. Effective screening for term PE was performed based on the Bayesian model with a detection rate of 75% at a screen-positive rate of 10% (Wright et al., 2020; Döbert et al., 2021). This model includes a combination of maternal factors with measures of mean arterial pressure (MAP) and the determination of serum placental growth factor (PlGF) and soluble fms-like tyrosine kinase-1 (sFlt-1). Maternal characteristics and obstetric history were collected from the medical records. Inclusion criteria for the study were as follows: age ≥18 years, singleton pregnancy, and a live fetus. Exclusion criteria were the existence of a major fetal anomaly, established PE, and congenital anomalies. Trial participants received written information, and those patients who agreed to participate in the study gave their written informed consent. The research was carried out in accordance with the ethical principles promulgated by the World Medical Association in the Declaration of Helsinki (1996) and in compliance with the local laws and regulations. Favorable authorization was received from the Committee for the Conduct of Research Work in Health Area I to the Area Management Board, dated 10 January 2019, and from the Experimental Biosafety Committee of the University of Murcia, dated 25 June 2019.

Of a total of 68 pregnant women at high risk of term PE, 35 women received a placebo and 33 women received a 20 mg/day dose of pravastatin for approximately 3 weeks, from 35 to 37 weeks of gestation until delivery. Pravastatin tablets and matched placebos were procured and over-encapsulated by Mawdsley-Brooks & Co. (Salford, United Kingdom). The placebo and pravastatin capsules were identical in parameters such as size, thickness, physical properties, and appearance. Mean arterial pressure, uterine artery pulsatility index, different serum markers of endothelial dysfunction, oxidative stress, and inflammation, and levels of circulating LEVs were measured before and after treatment in both groups of women. The data collected were assessed and recorded at follow-up clinic visits at 35–37 and 38–39 weeks of gestation. None of the participants developed PE during data collection.

MAP was determined using validated devices and standardized protocols (Poon et al., 2012). Uterine arteries were visualized through transabdominal color Doppler ultrasonography, where the pulsatility index of the right and left arteries was obtained and its mean value was calculated.

### Serum biomarkers

Lipid profile, uric acid (UA), and high-sensitivity C-reactive protein (hs-CRP) were determined by automated methods on a cobas c8000 modular platform (Roche Diagnostics^®^ International Ltd., Rotkreuz, Switzerland). Apolipoprotein B (ApoB), apolipoprotein A-I (ApoA-I), lipoprotein A (LpA), and homocysteine (HCy) concentrations were determined by immunonephelometry on a BN ProSpec compact autoanalyzer (Siemens Healthcare Diagnostics^®^, Margurg, Germany). Soluble fms-like tyrosine kinase-1 (sFlt-1) and placental growth factor (PlGF) determinations were performed using a fully automated B-R-A-H-M-S KRYPTOR Compact Plus system (Thermo Fisher Scientific).

Total plasma antioxidant capacity (TAC) was determined using a total antioxidant status assay kit (Cat. 615700, Sigma-Aldrich). Interleukin 6 (IL-6) and growth differentiation factor 15 (GDF-15) levels were determined by electrochemiluminescence immunoassay using a cobas e 411 analyzer (Roche Diagnostics^®^). The determination of asymmetric dimethylarginine (ADMA) was performed by competitive-inhibition enzyme-linked immunosorbent assay (cELISA) (Cat. No. KSB301Ge11, Cloud-Clone Corporation).

### LEV isolation, characterization, and quantification

Peripheral blood was collected from the cubital vein in citrated tubes and processed within 1 h to avoid the generation of *ex vivo* EVs. The samples were centrifuged at 3000 g for 5 min at room temperature, and then the plasma was carefully collected, leaving an unaltered layer of 1 or 2 mm at the interface with the blood cells. Aliquots of 400 µL were then made and frozen at −80°C until further analysis. The samples and patient data included in this study were preserved by the Biobank Network of the Region of Murcia (PT17/0015/0038), integrated into the National Biobank Network (B.000859), with the approval of the Ethics and Scientific Committees, and were processed following standardized procedures.

LEVs were characterized by flow cytometry with annexin V (AV) and fluorochrome-conjugated antibodies directed against cell-surface protein markers using an LSRFortessa X-20 flow cytometer (BD Biosciences) equipped with FACSDiva software. The isolation and characterization of LEVs were performed as previously described with certain modifications (Nieuwland et al., 1997; Suades et al., 2013; Witwer et al., 2013; Inglis et al., 2015.) and following the recommendations of MISEV2018 (Théry and Witwer, 2018).

Plasma aliquots were thawed for 1 hour and centrifuged at 3000 g for 10 min to remove the artifacts produced during storage. The LEV fraction was then isolated from the plasma by two-step high-speed centrifugation using a Sorvall ST 16 R Centrifuge (Thermo Scientific) with a fixed angle rotor (k = 483, k_adj_ = 660). Briefly, 250 µL of the plasma was centrifuged at 18890 g for 30 min to pellet the MVs. Then, 225 µL of the supernatant was discarded, and the LEV-enriched pellet was washed with PBS–citrate solution (1.4 mmol/L phosphate, 154 mmol/L NaCl, and 10.9 mM trisodium citrate, pH 7.4). A second centrifugation was performed under the same conditions, after which 225 µL of the supernatant was removed and the pellet was reconstituted in PBS–citrate solution to reach a final volume of 100 µL.

For direct labeling, 15 µL of isolated MVs was mixed with 35 µL of AV binding buffer (10 mM HEPES/NaOH, pH 7.4, 140 mM NaCl, and 2.5 mM CaCl_2_) and a master mix of FITC-conjugated AV (BD Pharmingen™ FITC annexin V) and fluorochrome-conjugated monoclonal antibodies against specific cell-surface antigens. Prior to EV staining, antibodies were filtered to remove aggregates that could cause unspecific signals. Thus, the antibody master mix was added into a tube containing a 0.22-µm nitrocellulose filter and centrifuged using a fixed angle rotor (750 × g) at room temperature for 2 min. Fluorochrome-conjugated antibodies used were anti-CD41a BV421 (BD Horizon™ Mouse Anti-Human CD41a, clone HIP8), against the anti-GpIIb receptor in platelets; anti-CD45 PerCP-Cy5.5 (BD PerCP Cy5.5 Mouse Anti-Human CD45, clone HI30), against the leukocyte common antigen; anti-CD33 APC (BD Pharmingen™ APC Mouse Anti-Human CD33, clone WM53), against sialic acid-binding Ig-like lectin 3, highly expressed in monocytes; anti-CD62E BV711 (BD Pharmingen™ APC Mouse Anti-Human CD62E, clone 68-5H11), against endothelial E-selectin; anti-CD54 BV650 (BD OptiBuild™ BV650 Mouse Anti-Human CD54, clone HA58), against intercellular adhesion molecule 1 (ICAM-1), expressed in both activated leukocytes and endothelium and anti-PLAP PE (Canvax PLAP Monoclonal Mouse Antibody, Cat. No. MA0074), and against placental alkaline phosphatase, in the syncytiotrophoblast. The samples were incubated for 15 min in the dark at room temperature and diluted with 650 µL of AV-binding buffer before acquisition. Quantification was carried out using TruCount Absolute Counting tubes (BD™ Catalog No. 340334.) containing a known number of standard beads.

**FIGURE 1:**
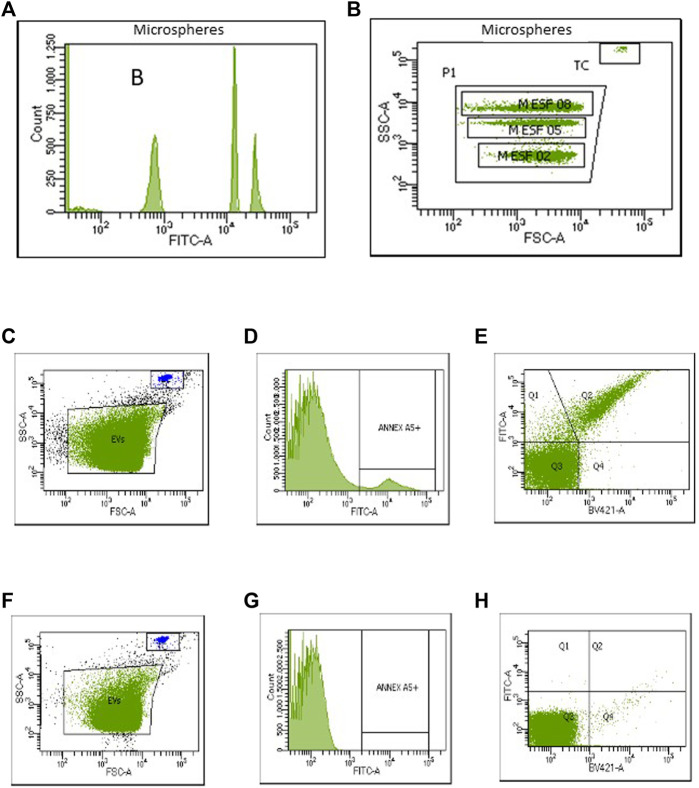
Flow cytometry resolution of a sizing bead and a large extracellular vesicle (LEV) gate using SSC as the main size parameter. **(A)** Histogram shows the resolution of 0.2 *µ*m, 0.5 *µ*m, and 0.8 *µ*m microspheres. **(B)** Same microspheres are depicted in the dot plot using SSC to set the LEV gate (P1). MESF, 0.2; 0.5; and 0.8 µm microspheres. The gate for capturing the counting beads (TC) is depicted on the dot plot. **(C–H)** Negative control study. After the first reading (panels C–E), 20 μL of 10% NP-40 was added to each sample, and then the acquisition was repeated (panels F–H) to allow the subtraction of positive events detected in the lysed sample.

The cytometer detection lower threshold was set above the electronic noise using a sample of filtered AV-binding buffer. Forward scatter, side scatter, and fluorescence voltage parameters were obtained daily by gain adjustments on the logarithmic scale using CompBead Plus Anti-Mouse Ig κ, BD™ CompBead Plus negative control, and the antibodies used in the study. To detect LEVs, a window was defined to collect events produced by LEVs with a diameter smaller than 1 μm, using a kit with calibrated microspheres of 0.2, 0.5, and 0.8 μm in diameter (Bangs Laboratories, Inc., Cat. No. 832) (Figure 1A). The compensation obtained for the panel was used in the setup of each experiment. EVs were identified according to their size, AV binding, and their affinity for cell origin-specific antibodies. Using these microspheres, we determined that the size of detected EVs in the defined window varied between 100 and 800 nm, with the majority of EVs being >200 nm; therefore, they were considered LEVs.

Acquisition was performed at a low flow rate until 10,000 events were counted in the TruCount bead window. The data were acquired using two or more parameter dot plots and histograms, taking into account the hierarchy of the panel. LEV phenotyping and origin were identified based on their FSC/SSC. To quantify the LEV concentration, the recorded events from each population were compared to those obtained from TruCount microspheres using the following equation:

LEVs = GLEV*TC/GTC*V, where GLEV was the number of events in the LEV gate, GTC is the number of events in the TruCount™ bead gate, and TC is the number of TruCount™ beads contained in the reaction tube.

**FIGURE 2:**
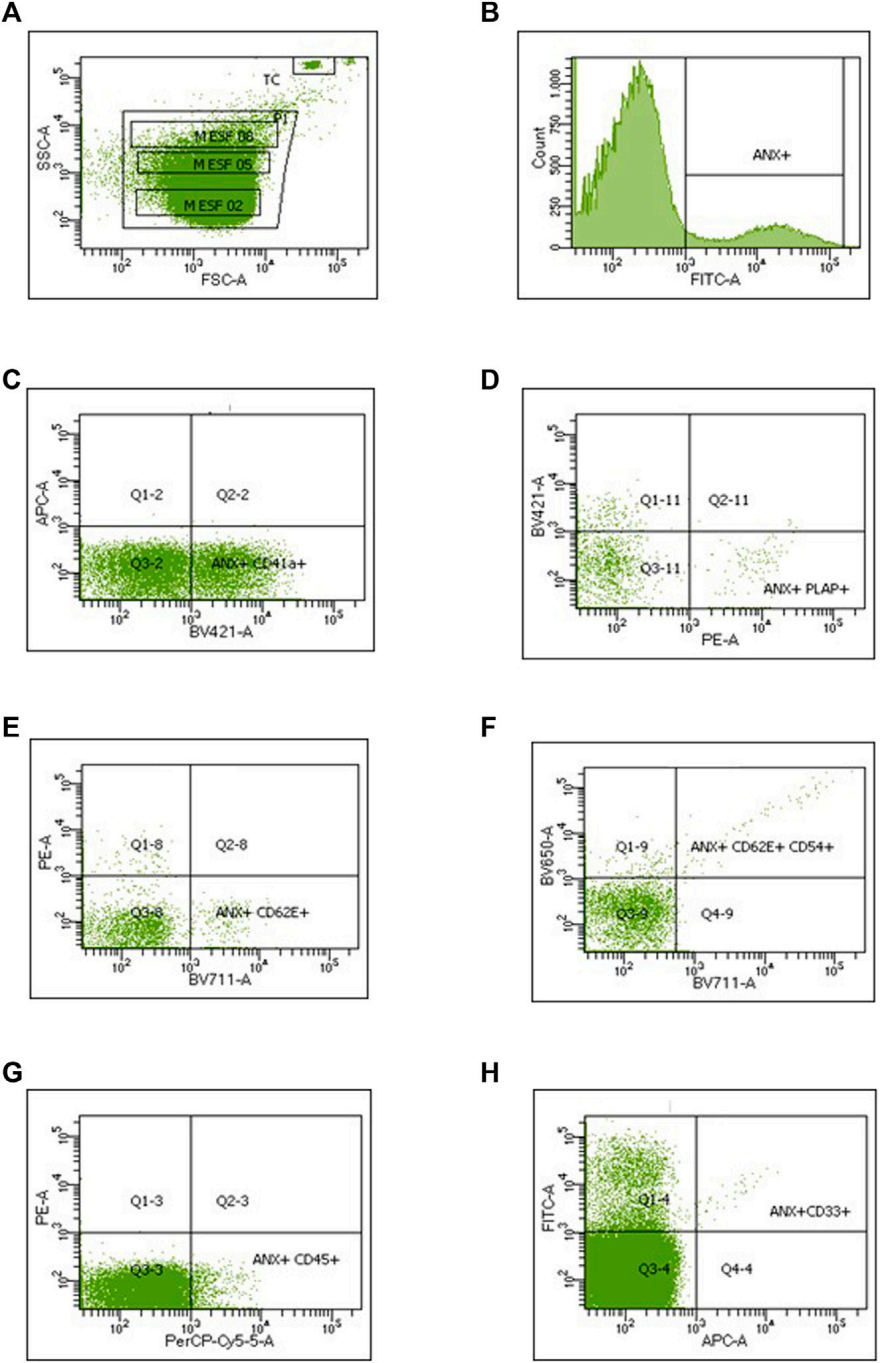
Dot plots and histograms obtained during the identification and quantification of large extracellular vesicles. **(A)** The P1 acquisition window was defined with calibrated microspheres of different diameters. Quantification was achieved by comparing the events recorded from each population or subpopulation of LEVs with those obtained from a known concentration of TruCount microspheres. TC, TruCount microspheres. **(B)** ANX, Annexin V^+^ (FITC-A). **(C)** Platelet-derived LEVs CD41a^+^/CD33^−^ (BV421-A vs*.* APC-A). **(D)** Syncytiotrophoblast-derived LEVs, PLAP^+^/CD41a^−^ (PE-A *vs.* BV421-A). **(E)** Endothelial-derived LEVs, CD62E^+^/PLAP^−^ (BV711-A vs*.* PE-A). **(F)** Activated endothelial-derived LEVs, CD54^+^/CD62E^+^ (BV711-A vs*.* BV650). **(G)** Leukocyte-derived LEVs CD45^+^/PLAP^−^ (PerCP-Cy5.5-A vs*.* PE-A). **(H)** Monocyte-derived LEVs CD33^+^ (APC-A vs*.* FITC-A).

The dot plot in Figure 2A shows the window defined for the acquisition of the LEVs (P1), as well as the area where the events produced by TruCount beads were counted. LEV populations were identified from AV + population (Figure 2B), according to their positive staining with the antibody of interest together with the absence of staining for another marker absent in the population (a representative assay is shown in Figures 2C–H). Positive or negative reactivity to specific antibodies is shown in Table 1. The ability of accurately assessing LEV phenotypes relies heavily on correct gating to separate positive events from background fluorescence; therefore, it is critical to choose a negative control that best appropriately mimics background fluorescence for a given sample. Lysed controls were preferred because they provide additional information about a sample (e.g., the presence of detergent-resistant, non-vesicle-related events, and/or aggregates) that can result in non-EV positive signals and improperly inflate antibody positive event counts. After the first reading (Figures 1B, C), 20 μL of 10% NP-40 was added to each sample, and then the acquisition was repeated to allow the subtraction of positive events detected in the lysed sample (Figures 1E–H).

**TABLE 1:** Antibody specificity and fluorochromes used to characterize LEVs.

LEV source	Antibody specificity	Fluorochrome	Positive marking
Platelets	CD41a	BV421	CD41a^+^/CD33^−^
Leukocytes	CD45	PerCP-Cy5.5	CD45^+^/PLAP^−^
Monocytes	CD33	APC	CD33^+^
Endothelium	CD62E	BV711	CD62E^+^/PLAP^−^
Activated endothelium	CD54 (ICAM-1)	BV650	CD54^+^/CD62E^+^
Syncytiotrophoblast	PLAP	PE	PLAP^+^/CD41a^−^

CD41a, platelet GpIIb; CD45, leukocyte common antigen; CD33, sialic acid binding Ig-like lectin 3; CD62E, E-selectin; CD54, ICAM-1 (intercellular adhesion molecule 1); PLAP, placental alkaline phosphatase.

### Statistical analysis

Statistical analysis was performed using SPSS for Windows, version 23 (Chicago, Illinois, United States). Qualitative variables were described according to their absolute frequency and percentage in each group or subgroup studied. The Kolmogorov–Smirnov test was applied to study the distribution of normality of the variables. Those variables showing a normal distribution were described by the mean and standard deviation of the mean. Those variables that did not follow a normal distribution were described by median and the interquartile range.

Qualitative variables were compared using the Chi-squared test, with Fisher’s exact test when relevant. To compare quantitative variables between two groups, the Mann–Whitney *U* test was used. The Wilcoxon signed-rank test was used for the analysis of related or paired samples. The Spearman’s correlation coefficient was used to study the association between quantitative variables. Student’s *t*-test was used to determine the differences between groups of variables with a normal distribution.

## Results

The characteristics of the study population are summarized in Table 2. A total of 68 pregnant women at high risk of term PE with a mean age of 32.8 years and mean pregnancy length of 35^+4^ weeks were recruited. None of the participants consumed alcohol or drugs. Among the total population of women at high risk of PE who participated in the study, 44% had a body mass index (BMI) >30 kg/m^2^ and 69% were primiparous. There was no difference between placebo- and pravastatin-treated groups in BMI, parity, height, family history of PE, and gestational age at the baseline.

**TABLE 2:** Characteristics of the participants.

Characteristic	Placebo	Pravastatin	Statistical significance (p)
	(*n* = 35)	(*n* = 33)	
*Demographical and epidemiological data*			
Age (years)	33.2 ± 6.3	32.4 ± 6.5	0.572
Weight (kg)	76.5 [70–85.4]	75 [70.2–84.9]	0.965
Height (m)	161 [157–164]	160 [157–167]	0.693
BMI (kg/m^2^)	30.2 ± 4.8	29.9 ± 4.6	0.632
White race	34 (97.7)	32 (97)	0.739
Smoking habit	5 (14.3)	0	0.031
Alcohol or drug use	0	0	—
Nuliparity	22 (62.9)	25 (75.8)	0.187
Family history of PE	3 (8.6)	5 (15.2)	0.321
*Comorbidities*			
Systemic erythematosus lupus	0	0	—
Antiphospholipid syndrome	1 (2.9)	0	0.515
Diabetes mellitus	1 (2.9)	0	0.515
Chronic arterial hypertension	1 (2.9)	1 (3)	0.739
*Pregnancy clinical data*			
Assisted reproduction	3 (8.6)	9 (25.7)	0.590
Gestational age	35.4 [35.3–35.7]	35.6 [35.3–35.7]	0.454
PE risk (1 in)	9 (6–14)	11 (5–15)	0.863
Mean arterial pressure (mmHg)	95.3 [91–100.7]	96.2 [91–99.8]	0.897
Mean arterial pressure (MoM)	1.07 ± 0.08	1.07 ± 0.07	0.883
Creatinine (mg/dL)	0.60 [0.51–0.66]	0.48 [0.44–0.59]	0.156
Proteinuria (mg/dL)	12 [10–79.5]	10 [6.75–18.5]	0.340
Platelets (×1,000/mm^3^)	215 [180–257]	194 [175–270]	0.676
Uterine artery PI	0.73 [0.61–0.9]	0.69 [0.6–0.8]	0.238
Uterine artery PI (MoM)	1.04 [0.85–1.3]	0.96 [0.84–1.13]	0.256
Gestational diabetes mellitus	5 (14.3)	3 (9.1)	0.753
Gestational-induced hypertension	7 (20)	4 (12.1)	0.381
Low-dose aspirin use	13 (37.1)	8 (24.2)	0.187
PE development	4 (11.4)	3 (9.1)	0.753
Labor			
Spontaneous	14 (40)	16 (48.5)	0.485
Induced	19 (54.3)	15 (45.5)	0.470
No labor	2 (5.7)	2 (6.1)	0.952
Vaginal	13 (37.1)	17 (51.5)	0.236
Cesarean	14 (40)	7 (21.2)	0.096
Instrumental	8 (22.9)	9 (27.3)	0.677
Gestational age at delivery (w)	39.7 [39.1–40.4]	39.9 [38.9–40.5]	0.749
Birth weight (g)	3,200 [2,760–3,555]	3,230 [3,012–3,473]	0.722

In our study population, 11% of the pregnancies at high risk of term PE developed gestational diabetes and 16% developed gestational hypertension, but there were no differences in the occurrence of these disorders in the two studied groups. Similarly, while 30% of the women included in the study had been previously treated with low-dose aspirin, the distribution between both groups was homogeneous. In addition, seven pregnant women developed PE, of which four belonged to the placebo group and three belonged to the group treated with pravastatin. Regarding pregnancy outcomes, labor started spontaneously in 44% of all women; it was induced in 12% and 31% were cesarean deliveries, with no differences between the two study groups. The gestational age at the time of delivery and newborn weights were similar in both groups.

The values of the biochemical parameters analyzed are shown in Table 3. As shown in the table, the group of women who received the placebo and those who were treated with pravastatin started from a similar baseline situation. In this sense, the lipid profile biomarkers studied showed no differences before treatment between the two groups. Treatment with pravastatin decreased total cholesterol, while the placebo had no effect. Triglyceride concentrations increased as pregnancy progressed in the placebo group but did not change after the administration of pravastatin. Similar to the total cholesterol, the ApoB and ApoB/ApoA-I ratio decreased significantly in the pravastatin-treated group, but they did not change as pregnancy progressed in the placebo group. However, total cholesterol LDL, ApoB, ApoB/ApoA-I, and LpA values were not significantly different between the placebo and pravastatin groups after treatment. There were no differences in proteinuria, serum creatinine, and platelet levels in any case before or after treatment between the groups.

**TABLE 3:** Changes in biomarkers in pregnant women at high risk of term PE treated with placebo or pravastatin.

Laboratory parameter	Pre-placebo	Post-placebo	Pre-pravastatin	Post-pravastatin
	(*n* = 35)	(*n* = 35)	(*n* = 33)	(*n* = 33)
*Dyslipidemia*				
Total cholesterol (mg/dL)	245 [200–245]	233 [203–264]	245 [214–278]	220 [187–251]*
Triglycerides (mg/dL)	268 [234–320.2]	290 [242–355]*	254 [205–289]	234 [188–283]
HDL (mg/dL)	66 [57.5–76.5]	63 [58–76]	68 [62–79]	71 [57–80]
LDL (mg/dL)	120.5 [86–145]	113 [82.5–132.5]*	122 [97–148]	99 [71–126]*
ApoB (mg/dL)	131 [109–147.7]	125 [110–150]	125 [117–162]	117 [95.5–135.5]*
ApoA-I (mg/dL)	219.5 [2.3–231.5]	218 [204–234]	213 [201–235]	222 [192.5–240.5]
ApoB/ApoA-I	0.58 [0.51–0.58]	0.6 [0.48–0.7]	0.59 [0.52–0.76]	0.52 [0.43–0.68]*
LpA (mg/dL)	10.5 [7.4–31.6]	9.9 [5.6–24.5]*	16.9 [7.6–32.7]	13.5 [5.8–31.5]
ADMA (ng/mL)	764 [434–1,631]	418 [345–1,610]	958 [598–1,474]	676 [452–1,323]
*Oxidative stress markers*				
TAC (mmol/L)	1.59 [1.55–1.66]	1.63 [1.6–1.69]*	1.58 [1.53–1.66]	1.62 [1.54–1.66]
HCy (µmol/L)	6.45 [5.67–7.22]	6.4 [5.6–7.7]	6.2 [5.7–6.9]	6.3 [5.65–7.3]
UA (mg/dL)	4.5 [4–5.1]	4.9 [4.3–5.9]*	4.7 [4–5.3]	4.9 [4.2–5.75]^+^
GDF-15 (µg/mL)	105.4 [74.7–151.2]	107.2 [77.1–165]	122.9 [77.6–182.8]	110.8 [82.3–184.2]
*Inflammation markers*				
IL-6 (pg/mL)	3.94 [2.65–4.85]	4.47 [3.5–5.58]	4.35 [3.04–5.57]	4.88 [3.61–5.87]
hs-CRP (mg/dL)	0.42 [0.26–0.70]	0.45 [0.21–0.84]	0.51 [0.27–0.73]	0.37 [0.25–0.78]
*Angiogenic markers*				
PlGF (pg/mL)	94.7 [55.9–125.7]	60.28 [48.35–88.7]*	91 [61.2–155.7]	59.8 [46.97–123.4]^+^
PlGF (MoM)	0.35 [0.23–0.52]	0.36 [0.19–0.56]	0.33 [0.25–0.7]	0.34 [0.30–1.87]
sFlt-1 (pg/mL)	5,119 [3,541.7–6,027.5]	6,934 [4,849–9,249]*	4,899 [3,693.5–6,516.5]	7,830 [5,930–9,289]*
sFlt-1 (MoM)	2.08 [ 1.63–3.06]	2.49 [1.71–3.07]	2.11 [1.68–2.75]	2.24 [0.99–2.74]
sFlt-1/PlGF	55.5 [31.87–95.37]	102.2 [74–188.1]*	59 [33.9–81.15]	116.5 [62.6–180.5]*

Data are shown as the median and interquartile. HDL, high-density lipoprotein cholesterol; LDL, low-density lipoprotein cholesterol; ApoB, apolipoprotein B; ApoA-I, apolipoprotein A-I; LpA, lipoprotein; ADMA, asymmetric dimethylarginine; TAC, total antioxidant capacity; HCy, homocysteine; UA, uric acid; GDF-15, growth/differentiation factor 15; IL-6, interleukin-6; hs-CRP, high-sensitivity C-reactive protein; PlGF, placental growth factor; sFlt-1, soluble fms-like tyrosine kinase 1; ^+^ *p* < 0.05; * *p* < 0.001.

The MAP of the pregnant women in the high-risk group did not undergo significant changes during the clinical trial. In those who received the placebo, the MAP at baseline was 95.3 [91–100.7] mmHg, a similar value to that measured at the end of the study, 95.8 [87.3–103.3] mmHg (*p* = 0.966). Participants of the pravastatin group showed a baseline MAP of 96.2 [91.1–99.8] mmHg, which decreased to 94.6 [91.7–100.1] mmHg after treatment, although this change was not statistically significant (*p* = 0.796).

No statistically significant differences were found in endothelial dysfunction, oxidative stress, inflammation, and angiogenesis biomarkers at the onset of the study. ADMA levels remained unchanged after the intervention in both groups. TAC increased in both arms of the study, being statistically significant (*p* < 0.001) only in the group that received the placebo. However, there was no difference between the groups at the end of treatment (*p* = 0.669). The plasma concentration of UA increased significantly in both groups, whether or not they received pravastatin, reaching similar values (*p* = 0.989). In contrast, the levels of HCy, GDF-15, and IL-6 did not change in any of the studied groups. Although hs-CRP levels were not significantly different after treatment in either group, women treated with pravastatin had lower values of this inflammatory marker. Regarding angiogenic markers, PlGF levels were significantly decreased in both paired serums of pregnant women at high risk of term PE who received the placebo and in those who received pravastatin, whereas sFlt-1 was increased in both arms of the trial. Furthermore, the sFlt-1/PlGF ratio changed in the two groups in the evaluated time window. Neither the PlGF MoMs of the pravastatin-treated group nor the sFlt-1 MoMs of either group changed significantly. There were no significant differences between the groups in regard to the treatment effects on PlGF, sFlt-1, and sFlt-1/PlGF ratio.

### Levels of circulating LEVs in response to pravastatin

**FIGURE 3:**
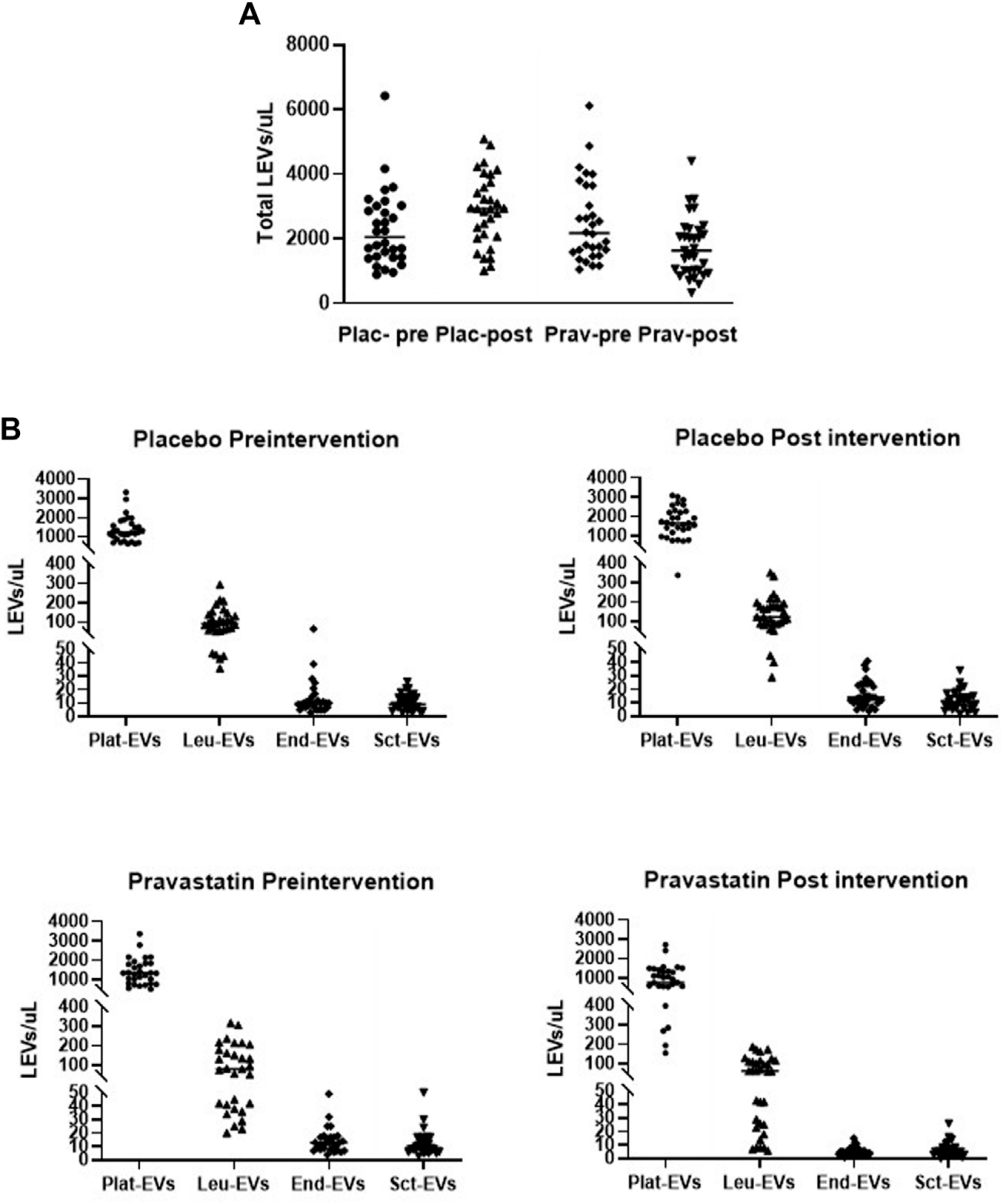
Comparison of large extracellular vesicle distribution and cellular origins between placebo- and pravastatin-treated pregnant women at high risk of term preeclampsia. **(A)** Scatter plots showing the total number of LEVs per microliter of the platelet-free plasma for each study patient in placebo (Plac-) and pravastatin (Prav-) groups before and after treatment. **(B)** Scatter plots showing the total number of LEVs per microliter of the platelet-free plasma from placebo and pravastatin groups by cell origin, before and after treatment. Plat-EVs, platelet-derived LEVs CD41a^+^/CD33^−^; Leu-EVs, CD45^+^/PLAP^−^ leukocyte-derived LEVs; End-EVs, CD62E^+^/PLAP^−^ endothelial-derived LEVs; Sct-EVs, PLAP^+^/CD41a^−^ syncytiotrophoblast-derived LEVs.

**FIGURE 4:**
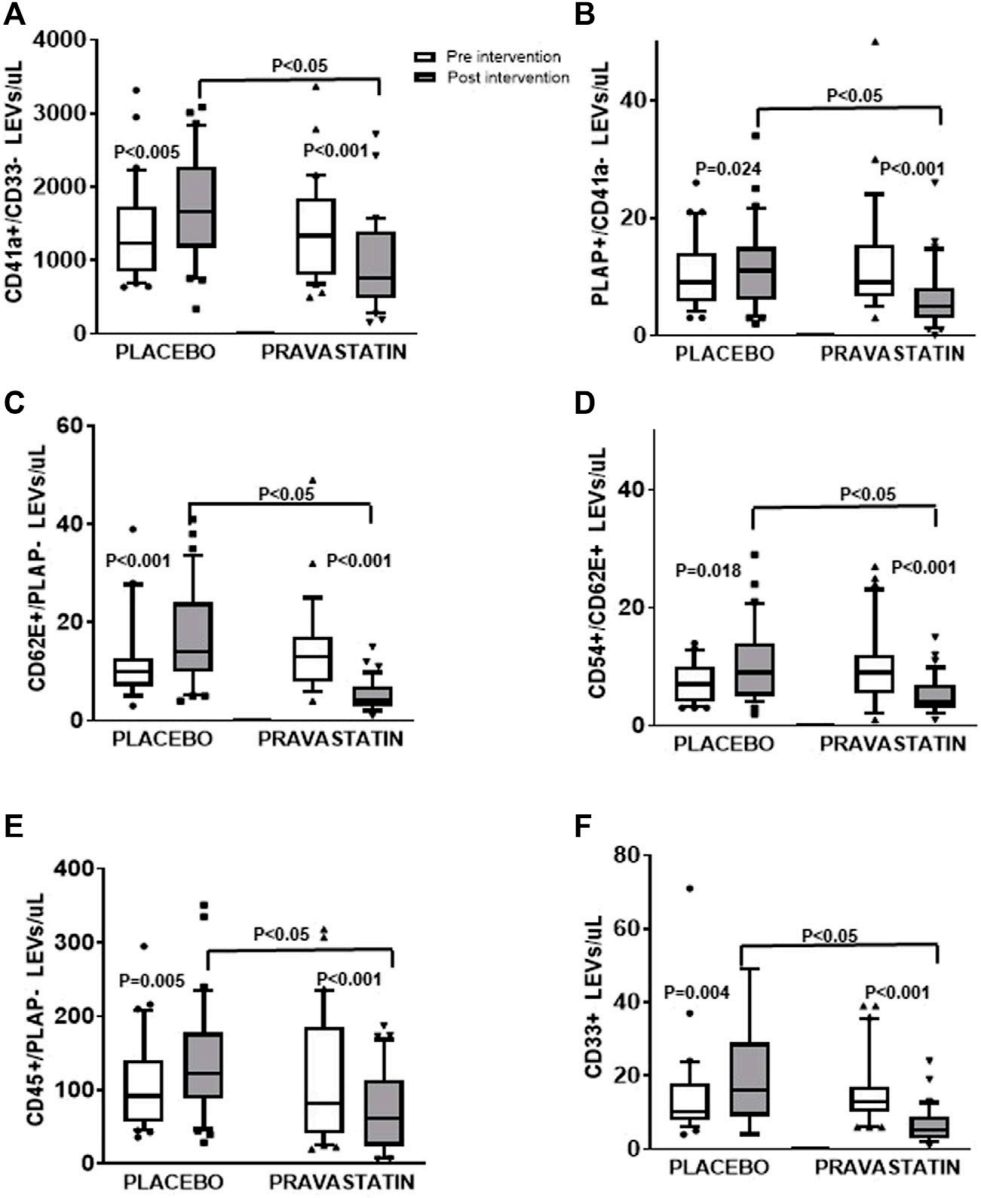
Effect of pravastatin on the plasma levels of large extracellular vesicle in pregnant women at high risk of term preeclampsia. Box and whisker plots show the number of LEVs per microliter of the platelet-free plasma, both before and after placebo or pravastatin treatment. Lines with boxes represent median values; the upper and lower box lines represent the 25th and 75th percentiles, respectively, and the upper and lower bars outside the boxes represent the 10th and 90th percentiles, respectively. Data points that are located outside the whiskers of the box plot represent the outliers, observations that are numerically distant from the rest of the data. **(A)** Platelet-derived LEVs CD41a^+^/CD33^−^. **(B)** PLAP^+^/CD41a^−^, syncytiotrophoblast-derived LEVs. **(C)** CD62E^+^/PLAP^−^, endothelial-derived LEVs. **(D)** CD54^+^/CD62E^+^, activated endothelial-derived MVs. **(E)** CD45^+^/PLAP^−^, leukocyte-derived LEVs. **(F)** CD33^+^, monocyte-derived LEVs.

Pravastatin treatment modified the plasma concentration of total EVs (AV+) in pregnant women at high risk of term PE. The concentration of total LEVs in the pregnant women who received the placebo rose from 2,048 [1,425–3,022] LEVs/µL to 2,931 [2,076–3,749] LEVs/µL (*p* = 0.008), while in those treated with pravastatin, there was a decrease from 2,153 [1,586–3,649] LEVs/µL to 1,637 [487–1,387] LEVs/µL (*p* < 0.001) (Figure 3A). Furthermore, after the intervention, the total LEV concentration was significantly lower in the group treated with pravastatin than the group treated with the placebo (*p* < 0.001) (Figure 3A). The scatter plot graphs in Figure 3B provide information about the distribution of LEVs before and after the intervention in the two arms of the study. Circulating LEVs showed a similar parental cell origin before treatment. Approximately 60% of the total LEVs were derived from platelets and 5% represented leukocytes and monocytes, whereas those derived from the endothelium and syncytiotrophoblast represented approximately 0.6% and 0.5%, respectively, in both groups studied. After treatment, whilst the percentage of platelet- and white cell type-derived LEVs did not change in both groups, there was a slight decrease in the percentages of endothelium- and syncytiotrophoblast-derived LEVs in the group of women treated with pravastatin. As shown in Figure 4, pregnant women at high risk of term PE, who received the placebo, showed a significant increase in the number of LEVs of various populations: platelet-derived LEVs (AV + CD41a^+^) increased by 34% (*p* = 0.005), those released by leukocytes (AV + CD45^+^) increased by 33% (*p* = 0.005), LEVs produced by monocytes (AV + CD33^+^) increased by approximately 60% (*p* = 0.004), LEVs of endothelial origin (AV + CD62E^+^) increased by 40% (*p* < 0.001), those of activated endothelium (AV + CD62E + CD54^+^) increased by 28% (*p* = 0.018), and syncytiotrophoblast-derived LEVs (AV + PAP^+^) increased by 22% (*p* = 0.024). In contrast, in the pravastatin-treated group, there was a significant decrease in the number of all types of LEVs, with the largest decrease being observed in those derived from the endothelium. In this way, total AV + LEVs decreased by approximately 24% (*p* < 0.001), platelet-derived LEVs decreased by 42% (*p* < 0.001), and leukocyte-released LEVs decreased by 25% (*p* < 0.001). In the same manner, the number of LEVs produced by monocytes dropped by 61% (*p* < 0.001) and endothelial LEVs and those derived from activated endothelium cells decreased by 69% (*p* < 0.001) and 55% (*p* < 0.001), respectively. Finally those shed from the syncytiotrophoblast showed a reduction of 44% (*p* < 0.001). As shown in Figure 4, when we compared the LEV levels from all studied origins, no significant differences were observed between the placebo and pravastatin groups at the time of pre-treatment. However, after treatment, the levels of all types of LEVs studied were significantly (*p* < 0.05) lower in the pravastatin-treated group than the placebo group.

### Time-dependent effects of pravastatin treatment

**FIGURE 5:**
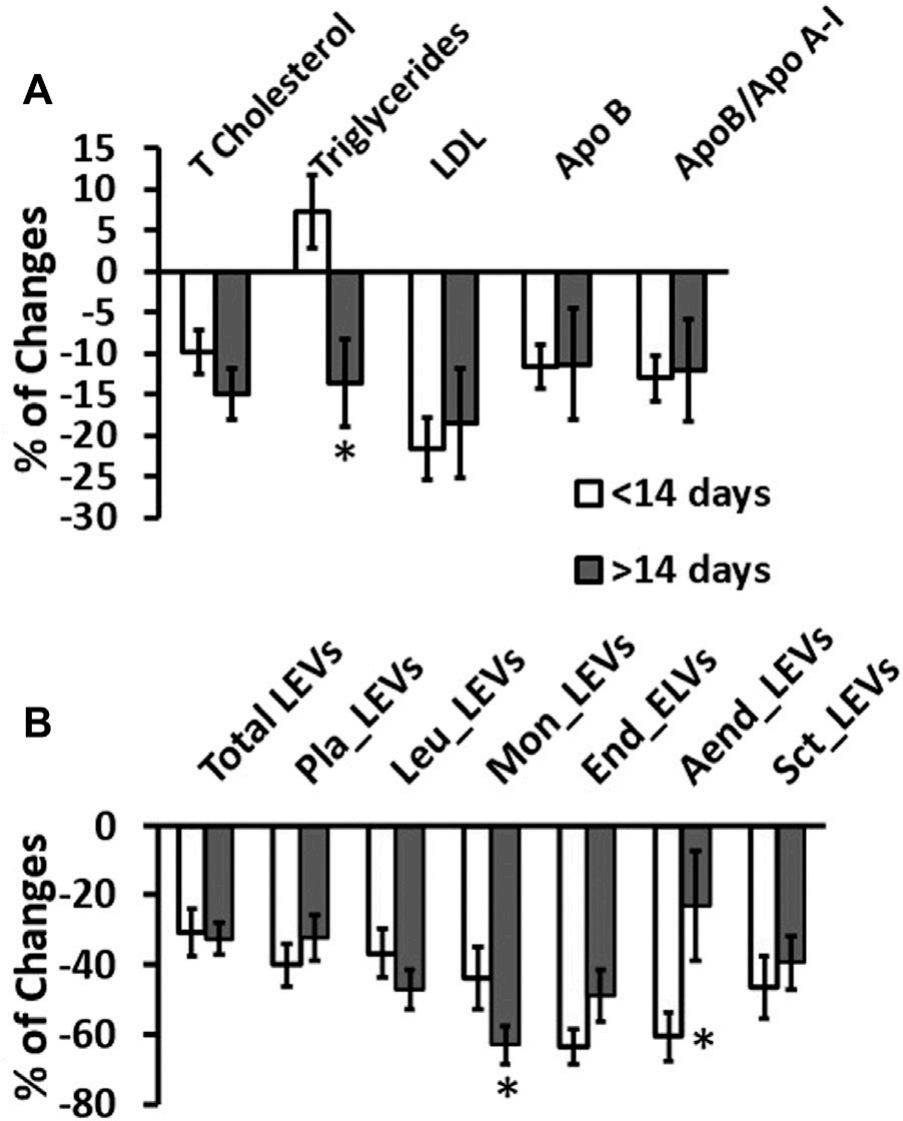
Changes in plasma biomarkers according to the time of pravastatin treatment. Bar plots represent % of change in plasma levels of lipidemic markers **(A)** and LEVs **(B)** related to the exposed days to pravastatin. *p <0.05 treatment >14 days vs*.* < 14 days.

The duration of treatment with pravastatin did not affect the levels of lipid profile markers (Figure 5A) and only a decreasing percentage trend was observed. However, the levels of LEVs from monocytes and activated endothelium cells were significantly lower in pregnant women treated for more than 14 days (Figure 5B). Furthermore, when comparing the percentage changes, we observed a significantly greater decrease in the levels of LEVs derived from endothelium and activated endothelium cells. In order to study the existence of an association between the hypolipidemic effect of pravastatin and the levels of LEVs, we performed a correlation between the levels of dyslipidemia markers that had undergone changes and those of the LEVs studied. We found no significant correlation, indicating a clear effect of pravastatin on vascular and inflammatory cell activation regardless of the plasma levels of dyslipidemia markers.

## Discussion

Women with a history of PE have an increased risk of developing hypertension, coronary heart disease, and other cardiovascular diseases in future (Boulanger Chantal et al., 2006; Sarker et al., 2014; Tannetta et al., 2017). The link between PE and cardiovascular diseases may be due to the shared risk factors such as dyslipidemia, obesity, hypertension, inflammation, oxidative stress, and endothelial dysfunction (Lok et al., 2008b; Salomon et al., 2016). In this regard, the results of a recent review summarizing the evidence for subclinical vascular dysfunction in women with prior hypertensive disorders of pregnancy compared with women with prior normotensive pregnancies (Zhang, 2022) suggest that the vascular damage sustained during hypertensive pregnancy persists after delivery and may contribute to future CVD development in these women. Therefore, the identification of these risk factors during gestation could be essential both for the prevention of potential gestational complications and for the early detection of future CVDs. A previous study by our group (Santoyo et al., 2022) demonstrated that women with a high risk of term PE, who did not develop the disease, showed higher MAP and elevated levels of different markers of oxidative stress, inflammation, and endothelial dysfunction compared with low-risk subjects. Furthermore, the levels of these markers were similar to those observed in women with high risk of term PE who developed the disorder. These results suggest that the existence of vascular dysfunction in non-developing PE high-risk pregnant women may also predispose them to a higher cardiovascular risk throughout life.

Statins have been the most commonly used drugs for the prevention of cardiovascular diseases. Although these drugs have been traditionally used to reduce plasma cholesterol levels, they also have pleiotropic effects that could be effective in the prevention and treatment of term PE. Many of the proposed pleiotropic actions of statins appear to be related to improving endothelial function by increasing nitric oxide bioavailability, reducing oxidative stress, and inhibiting inflammatory responses (Liao and Laufs, 2005), all of which could contribute to lowering the risk of developing PE at term and future CVDs in pregnant women. Taking these effects into account and considering several sources of evidence confirming that EVs are engaged in imbalanced angiogenesis, intravascular inflammation, and endothelial dysfunction and activation (Heng et al., 2021), we proposed the analysis of the effects of pravastatin on circulating LEV levels in women at high risk of term PE. The results of our study demonstrate that the women at high risk of term PE treated with pravastatin have a lower number of LEVs than those who received the placebo, suggesting an effect of this statin in reducing cell activation.

In the group of women at high risk of PE treated with the placebo, we found a significant increase in the plasma levels of LEVs of different cellular origins, from 35 to 37 weeks of gestation until the end of the study. We analyzed the concentration of each type of LEV separately, and we found that this group of pregnant women showed an increase in the levels of circulating endothelial LEVs expressing E-selectin (CD62E) and ICAM-1 (CD54) on their surface in the later stage of pregnancy, which reflects the activation of endothelial cells and endothelial dysfunction (Jiménez et al., 2003; Zhang, 2022; Mezine et al., 2023). The levels of endothelium-derived LEVs observed in the present study were similar to those observed in women with PE in the third trimester of pregnancy (Zelinka-Khobzey et al., 2021). In this sense, it has been shown that an increased content of circulating endothelial EVs in the blood of women with PE indicates endothelial damage, and the count of this type of EVs in the blood may serve as a marker for the severity of endothelial dysfunction in pregnant women (Zelinka-Khobzey et al., 2021). Moreover, increased levels of endothelial LEVs in the group of women treated with the placebo were also accompanied by an elevation in the plasma concentration of LEVs derived from syncytiotrophoblasts, platelets, monocytes, and other leukocytes, at the end of pregnancy. These results are in line with other published studies that have associated different risk factors for PE with the increased concentration of EVs derived from endothelial cells, leukocytes, and platelets. In this sense, factors such as obesity, history of chronic hypertension, diabetes mellitus, adverse pregnancy history, and autoimmune diseases, which have been taken into account to determine the risk of PE in our study, have also been related with an elevated number of MVs in non-pregnant women (Tramontano et al., 2010; Nielsen et al., 2011; Lacroix et al., 2012; Stepanian et al., 2013; Zhang et al., 2014).

According to the literature, platelet-derived LEVs were the most abundant LEV type found in the circulation of pregnant women with a high risk of PE included in our study. Furthermore, as we have previously mentioned, their levels became higher at the end of pregnancy in the group of women treated with the placebo. Although different studies have observed either reductions (Lok et al., 2008b; Marques et al., 2012), increases, or the absence of changes in the plasma concentration of platelet-derived MVs in women with PE (Meziani et al., 2006; Alijotas-Reig et al., 2012), elevated levels of this type of EV have been reported in patients with various cardiovascular diseases, including hypertension, diabetes, and coronary artery disease (Gkaliagkousi et al., 2019; Voukalis et al., 2019; Gkaliagkousi et al., 2021; Lazaridis and Gavriilaki, 2022). It has been demonstrated that platelet-derived MVs promote inflammation, thrombosis, and oxidative stress and are able to decrease nitric oxide production (Brambilla et al., 2022). They have been also reported to play a role in mediating adhesive interactions of monocytes with endothelial cells, activating the endothelium, increasing monocyte chemotaxis, and promoting the inflammatory process (Barry et al., 1998). In this regard, these types of MVs, together with leukocyte-derived MVs have been implicated in the release of several endothelial cytokines (Mesri and Altieri, 1998). In the current study, women at high risk of term PE treated with a placebo showed a significant increase in the levels of LEVs of monocytic and leukocytic origins at the end of pregnancy, suggesting that these EVs along with those derived from platelets could be involved in inflammatory processes and subsequent endothelial dysfunction in these subjects. This hypothesis is supported by several studies that have found elevated levels of leukocyte- and monocyte-derived MVs in disorders associated with endothelial dysfunction, including PE (Meziani et al., 2006; Lok et al., 2008b) and several cardiovascular diseases (Suades et al., 2014).

Additionally, women at high risk of term PE of the placebo group also showed an increase in circulating levels of syncytiotrophoblast-derived LEVs at the end of pregnancy, which reflects an enhanced release of LEVs from the placenta. Excessive shedding of placenta-derived extracellular vesicles often indicates placental pathologies, and it has been demonstrated that the intravenous infusion of EVs derived from injured placentas causes a PE-like condition in pregnant mice (Han et al., 2020). In this context, we also previously observed (Santoyo et al., 2022) that women at high risk of term PE showed elevated sFlt-1/PlGF ratios, a marker of placental dysfunction indicating an imbalance in pro-angiogenic and anti-angiogenic factors derived from the placenta (Rana et al., 2022). In addition, in the present study, the sFlt-1/PlGF ratio is even higher at the end of pregnancy in these subjects, which could suggest a greater degree of placental stress and dysfunction (Mitlid-Mork et al., 2022) in the women at high risk of late PE. Therefore, considering that syncytiotrophoblast-derived MVs affect the functions of maternal cells, including platelets, leukocytes, and endothelial cells (Cockell et al., 1997; Messerli et al., 2010; Tannetta et al., 2015; Villalobos-Labra et al., 2022), an increase in the circulating levels of these EVs derived from the malfunctioning placenta may be contributing to the endothelial activation and endothelial dysfunction in women at high risk of term PE. Finally, increments in the plasma concentration of UA could also be involved in the enhanced levels of endothelial LEVs found at the end of pregnancy in the group of women treated with the placebo, since it has been reported that hyperuricemia induces the release of endothelial MVs through the activation of endothelial cells *in vitro* in a concentration-dependent manner (Yu et al., 2021).

All these findings indicate that women at high risk of term PE may have endothelial dysfunction, and therefore an increased risk of future CVDs, even if they do not develop the disease during gestation. However, unlike early PE, term PE cannot be predicted by first trimester screening or prevented by prophylactic aspirin administration in this gestational stage (Santoyo et al., 2022). Effective screening for this late manifestation of the disease is provided by a combination of maternal factors with measurements of mean arterial pressure, serum PlGF, and sFlt-1 (Döbert et al., 2021). In recent years, statins have been considered to be potentially beneficial pharmacological interventions for the treatment of this type of PE. Recent clinical studies have evaluated the safety of statins, specifically pravastatin and its efficacy in the prevention of PE in high-risk pregnant women, leading to inconclusive results (Ahmed et al., 2020; Constantine et al., 2021; Döbert et al., 2021). In our study, the administration of pravastatin at a dose of 20 mg daily from 35 to 37 weeks of gestation until delivery did not reduce the incidence of term PE; however, it decreased all types of circulating LEVs in the absence of effects on mean arterial pressure and different markers of oxidative stress, inflammation, and angiogenesis. These results are consistent with several studies reporting beneficial effects of statins in lowering the enhanced levels of MVs of different origins, associated with hypertension (Oggero et al., 2022), hypercholesterolemia (Suades et al., 2013), ischemic cardiomyopathy (Huang et al., 2012), diabetes, and chronic kidney disease (Inglis et al., 2015). In the current study, the largest decreases in the levels of LEVs were observed in those derived from the activated endothelium cells, suggesting that a low dose of pravastatin administered daily from 35 to 37 weeks of gestation until delivery points to a beneficial effect on endothelial function in pregnancies at a high risk of late PE. In addition, it appears that pravastatin in our study has a clear effect on reducing vascular and blood cell shedding that is independent of its effects on cholesterol levels because even though the administration of statins decreased the total cholesterol and the ApoB/ApoA-I ratio, no correlation was found between the plasma levels of dyslipidemia markers and the concentration of LEVs. It has been shown that non-lipid-dependent statin effects may be mediated through their actions on the assembly of cytoskeleton components, whose integrity is essential for MV release by cells (van Niel et al., 2018). In this regard, treatment with statins has been shown to inhibit cytokine production and the activation of NADPH oxidase as a result of the disruption of small G-protein function, necessary to maintain cytoskeletal processes and extensions (Cordle and Landreth, 2005). On the other hand, the decrease in the plasma concentration of LEVs induced by pravastatin treatment was not accompanied by changes in the levels of angiogenic factors or in the sFlt-1/PlGF ratio. These results are in line with those obtained in the double-blind, placebo-controlled clinical trial of 1,120 women with pregnancies at high risk of term PE (Döbert et al., 2021), which our study was framed around. This study found neither the benefit of pravastatin at a daily dose of 20 mg in reducing the incidence of PE nor did it have an effect on the adverse outcomes or the serum levels of sFlt-1 and PlGF. Similarly, the administration of a higher dose, 40 mg, from 24 to 31 weeks of gestation in women with an early onset of PE was not associated with significant reductions in the sFlt-1/PlGF ratio (Ahmed et al., 2020). A further study conducted on women with a high risk of early PE has demonstrated that the administration of 20 mg of pravastatin starting at 12–17 weeks of gestation did not cause any change in the angiogenic profile of these subjects, although it reduced the rate of PE (Constantine et al., 2021). In view of all these results, we can suggest the possibility that an earlier onset of the treatment or a longer duration could be more effective in preventing the development of PE, regardless of its effect on restoring circulating levels of angiogenic and anti-angiogenic factors. In this sense, we observed that while the duration of treatment with pravastatin did not affect the levels of lipid profile markers, the levels of LEVs derived from leukocytes, endothelial cells, and activated endothelial cells were significantly lower in pregnant women treated for more than 14 days with 20 mg of pravastatin, suggesting that women with a high risk of term PE with a longer treatment period would have better endothelial function. We cannot rule out that factors other than the imbalance between angiogenic and anti-angiogenic factors may be contributing to endothelial damage in women at high risk of term PE at the end of gestation.

In summary, the results in this study have demonstrated that a low dose of pravastatin administered to women at high risk of term PE, from 35 to 37 weeks until delivery, blocked the increase in the circulating levels of LEVs of different cellular origins at the end of pregnancy and further decreased all types of vesicles, with a greater effect on those derived from the activated endothelial cells. These results indicate that although the administration of pravastatin did not decrease the incidence of late PE in our study, the duration of treatment and the dose used were sufficient to improve endothelial function in pregnant women at an increased risk of developing the disease. These findings are particularly relevant because they suggest that the administration of this statin to pregnant women at high risk of term PE, even if they do not develop the disease, could protect them from the later development of cardiovascular diseases. Further studies are needed to understand the contribution of changes in the number of EVs to vascular damage associated with PE risk factors and the beneficial effects of statins acting at this level.

## Data Availability

The raw data supporting the conclusion of this article will be made available by the authors, without undue reservation.
